# The expression signature of cancer‐associated KRAB‐ZNF factors identified in TCGA pan‐cancer transcriptomic data

**DOI:** 10.1002/1878-0261.12407

**Published:** 2019-02-16

**Authors:** Marta Machnik, Rafał Cylwa, Kornel Kiełczewski, Przemysław Biecek, Triantafillos Liloglou, Andrzej Mackiewicz, Urszula Oleksiewicz

**Affiliations:** ^1^ Department of Cancer Immunology Poznan University of Medical Sciences Poland; ^2^ Department of Diagnostics and Cancer Immunology Greater Poland Cancer Centre Poznan Poland; ^3^ Faculty of Mathematics, Informatics, and Mechanics University of Warsaw Warszawa Poland; ^4^ Faculty of Mathematics and Information Science Warsaw University of Technology Warszawa Poland; ^5^ Department of Molecular and Clinical Cancer Medicine University of Liverpool UK

**Keywords:** breast cancer, cancer, epigenetics, KRAB‐ZNF, lung cancer, TCGA

## Abstract

The KRAB‐ZNF (Krüppel‐associated box domain zinc finger) gene family is composed of a large number of highly homologous genes, gene isoforms, and pseudogenes. The proteins encoded by these genes, whose expression is often tissue‐specific, act as epigenetic suppressors contributing to the addition of repressive chromatin marks and DNA methylation. Due to its high complexity, the KRAB‐ZNF family has not been studied in sufficient detail, and the involvement of its members in carcinogenesis remains mostly unexplored. In this study, we aimed to provide a comprehensive description of cancer‐associated KRAB‐ZNFs using publicly available The Cancer Genome Atlas pan‐cancer datasets. We analyzed 6727 tumor and normal tissue samples from 16 cancer types. Here, we showed that a small but distinctive cluster of 16 KRAB‐ZNFs is commonly upregulated across multiple cancer cohorts in comparison to normal samples. We confirmed these observations in the independent panels of lung and breast cancer cell lines and tissues. This upregulation was also observed for most of the KRAB‐ZNF splicing variants, whose expression is simultaneously upregulated in tumors compared to normal tissues. Finally, by analyzing the clinicopathological data for breast and lung cancers, we demonstrated that the expression of cancer‐associated KRAB‐ZNFs correlates with patient survival, tumor histology, and molecular subtyping. Altogether, our study allowed the identification and characterization of KRAB‐ZNF factors that may have an essential function in cancer biology and thus potential to become novel oncologic biomarkers and treatment targets.

**Abbreviations**
ACC: adrenocortical carcinoma
BLCA: bladder urothelial carcinoma
BRCA: breast invasive carcinoma
CESC: cervical squamous cell carcinoma and endocervical adenocarcinoma
CHOL: cholangiocarcinoma
COAD: colon adenocarcinoma
DLBC: lymphoid neoplasm diffuse large B‐cell lymphoma
ESCA: esophageal carcinoma
GBM: glioblastoma multiforme
HNSC: head and neck squamous cell carcinoma
KAP1: KRAB‐associated protein‐1
KICH: kidney chromophobe
KIPAN: pan‐kidney cohort
KIRC: kidney renal clear cell carcinoma
KIRP: kidney renal papillary cell carcinoma
KRAB‐ZNF: Krüppel‐associated box domain zinc finger
LGG: brain lower grade glioma
LIHC: liver hepatocellular carcinoma
LUAD: lung adenocarcinoma
LUSC: lung squamous cell carcinoma
OV: ovarian serous cystadenocarcinoma
PAAD: pancreatic adenocarcinoma
PCPG: pheochromocytoma and paraganglioma
PRAD: prostate adenocarcinoma
READ: rectum adenocarcinoma
SARC: sarcoma
STAD: stomach adenocarcinoma
STES: stomach and esophageal carcinoma
TCGA: The Cancer Genome Atlas
TGCT: testicular germ cell tumors
THCA: thyroid carcinoma
THYM: thymoma
TRIM28: Tripartite‐motif containing 28
UCEC: uterine corpus endometrial carcinoma
UCS: uterine carcinosarcoma
UVM: uveal melanoma

## 1 Introduction

Carcinogenesis is a complex process in which normal cells acquire pathologic properties, such as sustained proliferation, disrupted apoptosis, dysregulation of metabolism, and escape from the immune system control (Hanahan and Weinberg, 2011). The process is driven mainly by the aberrant functioning of mutated genes. However, epigenetic changes, such as DNA methylation and histone modifications, play a pivotal role in the initiation and progression of cancer. Overall, the DNA methylation level in cancer cells is decreased, which can contribute to the activation of oncogenes. Simultaneously, tumor suppressor genes (TSGs) can be inactivated by promoter hypermethylation (Llinas‐Arias and Esteller, 2017). Alterations to the DNA methylation profile frequently correlate with changes affecting chromatin state. CpG island hypermethylation in cancer cells is associated with a decrease in histone active marks: histone H3 and H4 acetylation, H3K4 trimethylation, and gain of repressive marks: H3K9me3 and H3K27me3 (Llinas‐Arias and Esteller, 2017). Moreover, the expression of many chromatin modifiers is often deregulated in the tumor (Dawson and Kouzarides, 2012). Yet, the exact mechanisms involved in the regulation of the epigenetic landscape in cancer cells remain poorly characterized.

Krüppel‐associated box domain zinc finger proteins (KRAB‐ZNFs) are the most abundant family of epigenetic repressors found only in tetrapod vertebrates (Lupo *et al*., 2013; Ma *et al*., 2014). The human genome harbors over 800 KRAB‐ZNF transcripts encoding functional proteins, splicing variants, and pseudogenes (Corsinotti *et al*., 2013; Huntley *et al*., 2006) that may be involved in development, metabolism, proliferation, and carcinogenesis (Ecco *et al*., 2017; Lupo *et al*., 2013). Upon binding to DNA, KRAB‐ZNFs trigger transcriptional repression through interaction with KAP1 (KRAB‐associated protein‐1), also known as TRIM28 (Tripartite‐motif containing 28). TRIM28 acts as a scaffold for a multimolecular entity comprising the heterochromatin protein 1 (HP1), the H3K9me3‐specific histone methyltransferase SETDB1, and the histone deacetylase‐containing complex NuRD. Together, these proteins silence transcription by triggering the formation of heterochromatin (Czerwinska *et al*., 2017; Ecco *et al*., 2017; Groner *et al*., 2010). Moreover, we and others have shown that at least in a stem cell context, KRAB‐ZNFs may mediate methylation of the DNA sequences adjacent to their binding motifs (Oleksiewicz *et al*., 2017; Quenneville *et al*., 2011; Wiznerowicz *et al*., 2007).

A growing number of reports indicate that certain members of the KRAB‐ZNF family are involved in various aspects of carcinogenesis. For example, *ZNF545* negatively controls cellular proliferation, and its expression is reduced in multiple malignancies due to promoter hypermethylation (Xiang *et al*., 2017; Xiao *et al*., 2014). A tumor suppressor role was also described for *ZBRK1* (Chen *et al*., 2015; Lin *et al*., 2010) and *ZNF307* (Liang *et al*., 2017). Another member of the KRAB‐ZNF family, *ZNF224*, was demonstrated to exert both tumor suppressor and oncogenic functions depending on the cellular context. Specifically, in a chronic myelogenous leukemia cell line, *ZNF224* was shown to augment the signaling of Wilms’ Tumor 1 (*WT1*), which is a known TSG that controls the expression of genes involved in differentiation, apoptosis, and cell cycle progression (Florio *et al*., 2010). In contrast, in bladder cancer and chronic lymphocytic leukemia, *ZNF224* serves as an oncogene that mediates enhanced cell growth and resistance to apoptosis and chemotherapy (Busiello *et al*., 2017; Cho *et al*., 2016; Harada *et al*., 2010). Oncogenic properties were also reported for *ZKSCAN3*. This KRAB‐ZNF is upregulated in bladder cancer, while its knockdown induces apoptosis and reduces the viability of cancer cells in *in vitro* and *in vivo* experiments (Kawahara *et al*., 2016). Interestingly, most reports demonstrate that KRAB‐ZNFs act as TSGs and only a few studies focus on their tumor promotion potential. It is highly likely, however, that more KRAB‐ZNF factors are involved in carcinogenesis. Thus, there is an urgent need to search for such genes and to systematically characterize their functions in a cancer setting.

In the current study, we aimed to identify and characterize KRAB‐ZNF factors specific to cancer by exploring the KRAB‐ZNF expression signature in multiple cancer types. To this end, we used the transcriptomic datasets for various patient cohorts deposited in The Cancer Genome Atlas (TCGA) repositories. Our analysis allowed for identification of a small but distinct cluster of cancer‐associated KRAB‐ZNFs, whose expression becomes commonly upregulated in many tumors. Furthermore, we showed that their expression correlates with various clinicopathological features, including patient survival, as well as histological and molecular subtypes. Such properties point toward a putative role of these KRAB‐ZNFs in tumor biology. If this is the case, KRAB‐ZNFs may become novel targets of anticancer therapies or biomarkers for clinical patient management. Nevertheless, such a hypothesis requires further experimental testing. Altogether, these observations provide grounds for future studies exploring KRAB‐ZNF functions related to carcinogenesis.

## 2 Materials and methods

### 2.1 TCGA datasets

RNA‐seq and clinicopathological data (data release: 2015‐11‐01) were downloaded from: http://gdac.broadinstitute.org/runs/stddata__2015_11_01/data/. A snapshot of the data from this release is available in the RTCGA package on Bioconductor 10.18129/B9.bioc.RTCGA website (https://www.bioconductor.org/packages/release/bioc/html/RTCGA.html). Counts for RNA‐Seq reads for each gene isoform were downloaded from the TCGA database (https://cancergenome.nih.gov/abouttcga/aboutdata/datalevelstypes). Gene isoforms listed on the official TCGA releases were used. The patient cohorts used were as follows: bladder urothelial carcinoma [BLCA, Normal (N): 19, Tumor (T): 408], breast invasive carcinoma (BRCA, N: 112, T: 1093), cholangiocarcinoma (CHOL, N: 9, T: 36), esophageal carcinoma (ESCA, N: 11, T: 184), head and neck squamous cell carcinoma (HNSC, N: 44, T: 520), kidney chromophobe (KICH, N: 25, T: 66), kidney renal clear cell carcinoma (KIRC, N: 72, T: 533), kidney renal papillary cell carcinoma (KIRP, N: 32, T: 290), liver hepatocellular carcinoma (LIHC, N: 50, T: 371), lung adenocarcinoma (LUAD, N: 59, T: 515), lung squamous cell carcinoma (LUSC, N: 51, T: 501), prostate adenocarcinoma (PRAD, N: 52, T: 497), stomach and esophageal carcinoma (STES, N: 15, T: 221), thyroid carcinoma (THCA, N: 59, T: 501), and uterine corpus endometrial carcinoma (UCEC, N: 11, T: 370). KRAB‐ZNF isoform expression across various tumor types was also tested in the additional cohorts, including: adrenocortical carcinoma (ACC, *n* = 79), cervical squamous cell carcinoma and endocervical adenocarcinoma (CESC, *n* = 304), colon adenocarcinoma (COAD, *n* = 285), COADREAD (*n* = 379), lymphoid neoplasm diffuse large B‐cell lymphoma (DLBC, *n* = 15), glioblastoma multiforme (GBM, *n* = 153), GBMLGG (*n* = 669), brain lower grade glioma (LGG, *n* = 516), ovarian serous cystadenocarcinoma (OV, *n* = 303), pancreatic adenocarcinoma (PAAD, *n* = 178), pheochromocytoma and paraganglioma (PCPG, *n* = 179), rectum adenocarcinoma (READ, *n* = 94), sarcoma (SARC, *n* = 259), SKCM (*n* = 103), stomach adenocarcinoma (STAD, *n* = 37), testicular germ cell tumors (TGCT, *n* = 150), thymoma (THYM, *n* = 120), uterine carcinosarcoma (UCS, *n* = 57), and uveal melanoma (UVM; *n* = 80). Complete names for each cohort are provided in the Abbreviations section.

### 2.2 Independent validation set of lung and breast cancer tissue samples

Lung cancer and matched normal tissues were collected from 58 patients operated on between 1995–2005 at the Liverpool Heart and Chest Hospital, UK. All patients provided informed consent, and ethical approval was obtained from the Liverpool Ethics Committee. The patient group represents a typical cohort with an operable lung tumor as decided according to standard treatment protocols. The samples were snap frozen and microdissected to ensure 80% cancer cell content. To test KRAB‐ZNF expression in breast cancer, we used commercially available Breast Cancer cDNA Array II purchased from Origene (Rockville, MD, USA) that also provided the clinical and molecular information of patients from whom samples were collected. The molecular data included ER, PR, and HER2 statuses of patients, assessed through immunohistochemistry. The tumors with weakly positive HER2 staining were also analyzed through the fluorescent *in situ* hybridization assay to detect HER2 amplification. The detailed characterization of clinicopathological and molecular data for lung and breast cancer cohorts used in this study is presented in Table 1. The expression level of the majority of KRAB‐ZNF factors remained low and sometimes undetected in our qPCR assays, which explains the differing sample numbers in the analyses for different factors.

**Table 1:** Clinical and histopathological parameters available for breast and lung cancer tumor cohorts

BRCA	No.	%	Lung	No.	%
Tumor stage			Tumor stage		
T1	14	32.6	T1	3	5.2
T2	20	46.5	T2	44	75.9
T3	3	7.0	T3	8	13.8
T4	2	4.7	T4	3	5.2
TX	4	9.3			
Nodal status			Nodal status		
Negative	16	37.2	Negative	27	46.6
Positive	20	46.5	Positive	31	53.4
NX	7	16.3			
Gender			Gender		
Male	0	0	Male	44	75.9
Female	43	100	Female	14	24.4
ER status			Differentiation		
Negative	10	23.3	Good	19	32.8
Positive	25	58.1	Moderate	31	53.4
Equivocal	1	2.3	Poor	7	12.1
No data	7	16.3			
PR status			Histology		
Negative	9	20.9	SqCCL	32	55.2
Positive	25	58.1	AdenoCa	24	41.8
Equivocal	2	4.6	Anaplastic Ca	1	1.7
No data	7	16.3			
HER2+			NSCLC	1	1.7
Negative	28	65.1	Follow‐up		
Positive	8	18.6	Alive	4	6.9
No data	7	16.3	Dead	52	89.7
Triple negative breast cancer	4	9.3	Age, mean age (range)	67.1	48.5–87.5

Data collected in The Human Protein Atlas (www.proteinatlas.org) (Uhlen *et al*., 2015) were used as another validation set. Immunohistochemical staining on tissue microarrays for lung cancer was performed on different numbers of samples for KRAB‐ZNF: ZNF205 (*n* = 12), ZNF320 (*n* = 10), ZNF485 (*n* = 11), ZNF643 (*n* = 11), ZNF695 (*n* = 9), ZNF707 (*n* = 11), and ZNF 789 (*n* = 12). There were no data available for ZNF273, ZNF525, and ZNF714. For breast cancer, the samples were available for: ZNF205 (*n* = 11), ZNF320 (*n* = 10), ZNF485 (*n* = 11), ZNF643 (*n* = 11), ZNF695 (*n* = 12), ZNF707 (*n* = 10), and ZNF789 (*n* = 12). There were no data for ZNF273, ZNF525, and ZNF714. Images are available at the following URL: ZNF205 BRCA (https://www.proteinatlas.org/ENSG00000122386-ZNF205/pathology/tissue/breast+cancer#img), LUNG (https://www.proteinatlas.org/ENSG00000122386-ZNF205/pathology/tissue/lung+cancer#img); ZNF320 BRCA (https://www.proteinatlas.org/ENSG00000182986-ZNF320/pathology/tissue/breast+cancer#img), LUNG (https://www.proteinatlas.org/ENSG00000182986-ZNF320/pathology/tissue/lung+cancer#img); ZNF485 BRCA (https://www.proteinatlas.org/ENSG00000198298-ZNF485/pathology/tissue/breast+cancer#img), LUNG (https://www.proteinatlas.org/ENSG00000198298-ZNF485/pathology/tissue/lung+cancer#img); ZNF643 BRCA (https://www.proteinatlas.org/ENSG00000187801-ZFP69B/pathology/tissue/breast+cancer#img), LUNG (https://www.proteinatlas.org/ENSG00000187801-ZFP69B/pathology/tissue/lung+cancer#img); ZNF695 BRCA (https://www.proteinatlas.org/ENSG00000197472-ZNF695/pathology/tissue/breast+cancer#img), LUNG (https://www.proteinatlas.org/ENSG00000197472-ZNF695/pathology/tissue/lung+cancer#img); ZNF707 BRCA (https://www.proteinatlas.org/ENSG00000181135-ZNF707/pathology/tissue/breast+cancer#img), LUNG (https://www.proteinatlas.org/ENSG00000181135-ZNF707/pathology/tissue/lung+cancer#img); ZNF789 BRCA (https://www.proteinatlas.org/ENSG00000198556-ZNF789/pathology/tissue/breast+cancer#img), LUNG https://www.proteinatlas.org/ENSG00000198556-ZNF789/pathology/tissue/lung+cancer#img.

### 2.3 Cell culture

All cell lines were cultured under standard conditions (37 °C, 5% CO_2_) in a humidified incubator. For breast cancer, we used nine different cell lines representing distinct molecular subtypes: luminal A (MCF7, T47D), luminal B (BT474), basal (BT20, BT549, HS578T, MDA‐MB231, MDA‐MB468), and HER2 positive (SKBR3). All breast cancer cell lines were cultured in DMEM, High Glucose (Biowest, Nuaillé, France) with 10% fetal bovine serum (FBS; Biowest), 1% penicillin/streptomycin (Gibco, Carlsbad, CA, USA), and 10 ng·mL^−1^ insulin (Sigma‐Aldrich, Darmstadt, Germany). As a normal control, we used mammary epithelial cell line MCF12A cultured in DMEM/F12 (Gibco) supplemented with 5% FBS (Biowest), 20 ng·mL^−1^ EGF (Sigma‐Aldrich), 0.5 ng·mL^−1^ hydrocortisone (Sigma‐Aldrich), 10 ng·mL^−1^ insulin (Sigma‐Aldrich), and 1% penicillin/streptomycin (Gibco). For lung cancer analysis, we used 2 lung fibroblast cell lines (IMR90 and LUNG14), 7 LUAD cell lines (A549, CALU3, CALU6, H1299, H2073, H358, and SKLU1), five LUSC cell lines (CALU1, HTB59, HTB182, LUDLU1, and SKMES), one small cell lung carcinoma cell line (DMS53), and one large cell lung carcinoma cell line (CORL23). All lung cancer and fibroblast cell lines were cultured in DMEM/F12 (Gibco) with 10% FBS (Biowest) and 1% penicillin/streptomycin (Gibco). As a normal control, we used NHBE (Normal Human Bronchial Epithelium) cells (Lonza CC‐2540) cultured in Bronchial Epithelial Growth Medium BulletKit (Lonza, Basel, Switzerland). All remaining cell lines were purchased from ATCC (Manassas, VA, USA).

### 2.4 RT‐qPCR expression analysis

For RT‐qPCR expression analysis, RNA was isolated with a Quick‐RNA™ MiniPrep kit (Zymo Research, Irvine, CA, USA) and converted to cDNA with an iScript kit (Bio‐Rad, Hercules, CA, USA) according to the manufacturers’ protocols. Gene expression analysis was performed using the LightCycler 480 SYBR Green Master Mix (Roche, Basel, Switzerland) with 5 μm primer mix on a LightCycler 96 machine (Roche). We selected endogenous control genes based on in‐lab testing and previously published literature: ACTB for BRCA samples (Maltseva *et al*., 2013) and ESD for lung samples (Gresner *et al*., 2009). Relative quantification was performed using the comparative Ct method (RQ = 2^−ΔΔCt)^. NHBE and MCF12A cell lines served as calibrators for expression analysis in lung and breast cancer samples, respectively. We used the following primers: ESD: FW 5′ – TGATCAAGGGAAAGATGACCA – 3′, RV 5′ – AACCCTCTTGCAATCGAAAA – 3′; ACTB: FW 5′ – CAGCCATGTACGTTGCTATCCAGG – 3′, RV 5′ – AGGTCCAGACGCAGGATGGCATG – 3′; ZNF205: FW 5′ – CAGAAGAAAAATGGGCTGTCA – 3′, RV 5′ – CGCCTCTCCACTCCTTCTC – 3′; ZNF273: FW 5′ – ACAGCTAAGACGCCAGGACT – 3′, RV 5′ – TGTGAAGTGTCCAGGCATTG – 3′; ZNF320: FW 5′ – CAGAGACGTGATGCTGGAGA – 3′, RV 5′ – TGCCCTGTTGATGACAATGTA – 3′; ZNF485: FW 5′ – GAGTGGAGACACCTGGATGC – 3′, RV 5′ – TTTTGGTTTGGAAGAGAGAAGC – 3′; ZNF525: FW 5′ – AGGGACGTGATGCTGGAG – 3′, RV 5′ – ATTGCCTTGTGCTGTTGATG – 3′; ZNF643: FW 5′ – GTGGGAGGATGTGACTAAGATGT – 3′, RV 5′ – ACTTTCGCCCTGGGTCTC – 3′; ZNF695: FW 5′ – ACAGAAACCTGATCTCCCTTG – 3′, RV 5′ – TTCACGTTCCAGGGCTCTTTC – 3′; ZNF707: FW 5′ – GAGTTCCAGGCAGTGCAGA – 3′, RV 5′ – CGTGGGTTTTCTTGTGAGCC – 3′; ZNF714: FW 5′ – GCCCTGGAATATGAAGATATG – 3′, RV 5′ – TTCTTAACTGTAAATTCTCATGT 3′; ZNF789: FW 5′ – GATGTGATGTTGGAGAACTACAGG – 3′, RV 5′ – CCAGGATCCACTGCTCGT – 3′.

### 2.5 Statistical analyses

RNA‐seq data downloaded from TCGA were normalized using RSEM (Li and Dewey, 2011), and differential analysis was performed using the DESeq package (Anders and Huber, 2010). To ensure sufficient sample size for the comparative expression analysis between normal and tumor tissues, we chose only those cohorts that contained at least nine normal samples. A heatmap was generated based on the results of the binomial test with a cutoff threshold of a median of absolute log_2_ fold change > 1/2. A *t*‐test with Tukey HSD correction was used to perform a comparative analysis of normalized KRAB‐ZNF expression between normal and tumor samples for BRCA, LUAD, and LUSC. *T*‐test with FDR correction was used to assess the differences between independent groups within clinical and molecular parameters. The statistical significance of changes in KRAB‐ZNF isoforms was calculated using a *t*‐test with FDR correction with pooled standard deviation. For the variant analysis, protein domains were assessed with the Expasy‐Prosite Database of Protein Domains, Families and Functional Sites Tool (https://prosite.expasy.org). The relationship between patient survival and KRAB‐ZNFs expression was tested with the log‐rank test and plotted with the Kaplan–Meier curves. KRAB‐ZNFs expression was transformed into binary high/low groups based on maximally ranked statistics. For the RT‐qPCR expression analysis, the difference in gene expression between normal and cancer tissues was calculated using the nonparametric Wilcoxon signed‐rank test for paired data (lung cancer) and Mann–Whitney *U*‐test for unpaired data (BRCA). All analyses described in the current study that utilized KRAB‐ZNF expression in TCGA datasets were deposited into an online application called KRAB‐ZNF Explorer – http://mi2.mini.pw.edu.pl:8080/KRAB_ZNF/ (R. Cylwa, K. Kiełczewski, U. Oleksiewicz, M. Machnik and P. Biecek, unpublished data). KRAB‐ZNF Explorer contains result tables and graphs that allow expression profiling of all KRAB‐ZNF factors in TCGA datasets, including gene and isoform expression in tumor and normal tissues, correlation with clinicopathological parameters, and association with CpG methylation.

## 3 Results

### 3.1 A subgroup of KRAB‐ZNF factors is overexpressed in 16 cancer types in TCGA datasets

**Figure 1:**
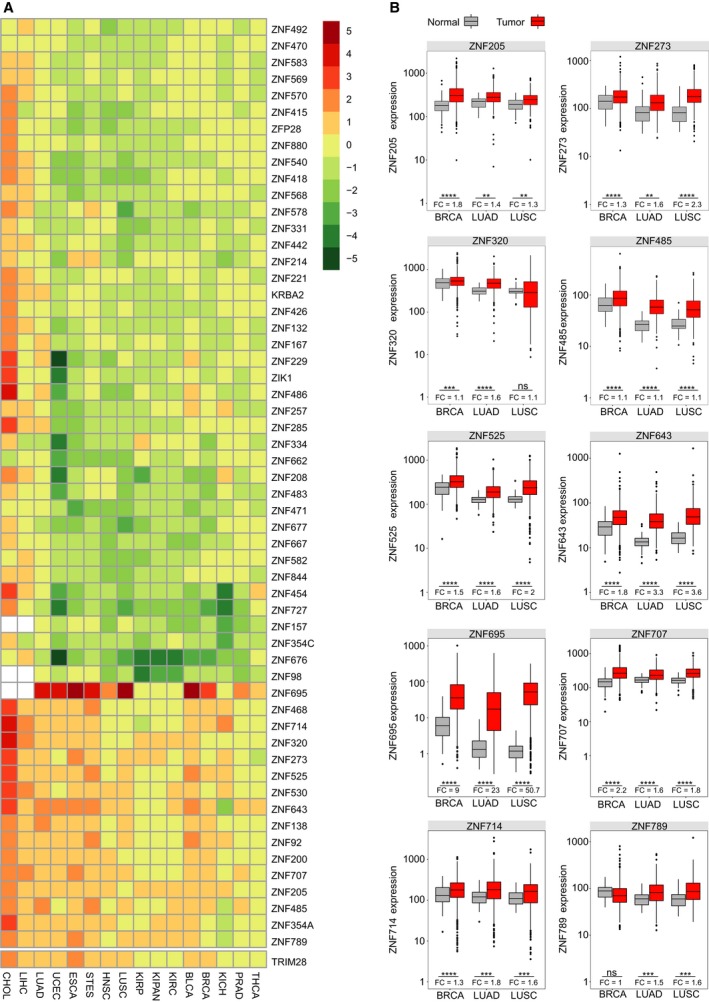
The expression level of selected KRAB‐ZNFs in TCGA samples. (A) Heatmap and supervised clustering of KRAB‐ZNFs with changed expression in 16 tumor cohorts compared to normal tissues. RSEM data were normalized with DESeq. Results were generated based on the binomial test with a cutoff threshold of a median of absolute log_2_ fold change > 1/2. (B) Boxplots representing selected cancer‐associated KRAB‐ZNF expression in normal and tumor tissue samples of breast and lung cancers. BRCA (N: 112, T: 1093), LUAD (N: 59, T: 515), LUSC (N: 51, T: 501). *P < 0.05, **P < 0.01, ***P < 0.001, ****P < 0.0001 as assessed by *t*‐test with Tukey HSD correction.

We first aimed to identify KRAB‐ZNFs that are commonly overexpressed in cancer cells. Since KRAB‐ZNF expression is frequently tissue‐specific, we compared the mRNA level of 381 human KRAB‐ZNFs (Corsinotti *et al*., 2013) between tumor and normal tissues using RNA‐seq profiling datasets collected in the TCGA project. For the statistical analysis, we removed the data for cancer types with a low number (*n* < 9) of corresponding normal tissues to ensure sufficient abundance of samples in each set. Thus, the data available for the analysis comprised 6727 tumor and normal tissue samples from 16 cancer types: BLCA, BRCA, CHOL, ESCA, HNSC, KICH, KIPAN, KIRC, KIRP, LIHC, LUAD, LUSC, PRAD, STES, THCA, and UCEC. Our differential expression analysis revealed that out of 381 KRAB‐ZNFs, only a fraction was deregulated in tumors (56 out of 381, 14.7%; with a cutoff = median of absolute log_2_ fold change > 1/2) (Fig. 1A). Interestingly, the majority of the KRAB‐ZNFs with an altered mRNA level exhibited reduced expression, while only a small but distinct cluster of 16 KRAB‐ZNFs showed upregulation in multiple cancer types (Fig. 1A). The upregulated KRAB‐ZNFs included: *ZNF695*,* ZNF468*,* ZNF714*,* ZNF320*,* ZNF273*,* ZNF525*,* ZNF530*,* ZNF643*,* ZNF138*,* ZNF92*,* ZNF200*,* ZNF707*,* ZNF205*,* ZNF485*,* ZNF354A*, and *ZNF789*. The highest significant fold change (FC) was observed for *ZNF695* in CHOL (54.9, *P* < 0.001). We also wanted to test the expression status of *TRIM28*, which encodes the KRAB‐ZNFs co‐factor protein, in the analyzed samples. As expected, *TRIM28* showed higher mRNA expression in the majority of analyzed tumors. The TRIM28/KRAB complex triggers epigenetic repression of specific target genes (Oleksiewicz *et al*., 2017) and thus might be involved in alterations occurring in cancer cells. Among all of the 240 comparisons of tumor versus normal tissues, 148 were statistically significant (61.7%). Within these 148 comparisons, significantly higher expression in normal tissues compared to tumor tissues was observed only in six cases (*ZNF138* in KIRC and THCA, *ZNF200* in THCA, *ZNF273* in PRAD, *ZNF485* in THCA, and *ZNF789* in KICH).

To investigate cancer‐associated KRAB‐ZNFs in more detail, we further narrowed down the analysis to the two most common cancer types: breast cancer (BRCA) and nonsmall cell lung cancer (adenocarcinoma: LUAD and squamous cell carcinoma: LUSC) (Siegel *et al*., 2017). We chose 10 KRAB‐ZNFs (*ZNF205*,* ZNF273*,* ZNF320*,* ZNF485*,* ZNF525*,* ZNF643*,* ZNF695*,* ZNF707*,* ZNF714*, and *ZNF789*) and looked closer at their expression profiles in the selected cancer types. First, we compared their expression level in normal and tumor tissues. We found that all of the cancer‐associated KRAB‐ZNFs were significantly upregulated in tumors compared to normal tissues in the TCGA RNA‐seq data (Fig. 1B). The highest difference in the expression was observed in the case of *ZNF695* (LUAD: FC = 23.0, *P* < 0.0001; LUSC: FC = 50.7, *P* < 0.0001; BRCA: FC = 9.0, *P* < 0.0001; *t*‐test with Tukey HSD correction). The smallest significant difference was noticed for *ZNF205* in lung cancer (LUAD: FC = 1.4, *P* < 0.01; LUSC: FC = 1.3, *P* < 0.01; *t*‐test with Tukey HSD correction) and *ZNF320* in BRCA (FC = 1.1, *P* < 0.001; *t*‐test with Tukey HSD correction).

### 3.2 The overexpression of selected KRAB‐ZNFs is confirmed in the independent validation sets of lung and breast cancer cell lines and tissues

**Figure 2:**
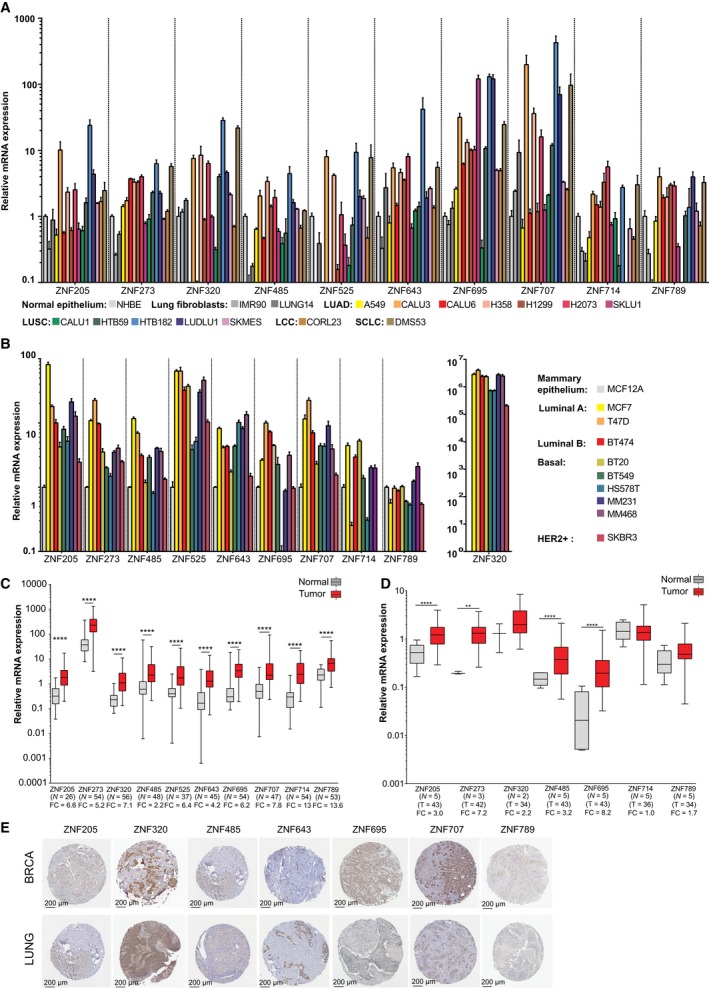
The expression level of selected KRAB‐ZNFs in independent sets of breast and lung cell lines and tissues. (A) RT‐qPCR expression of selected KRAB‐ZNFs in lung cancer cell lines compared to normal human bronchial epithelium. (B) RT‐qPCR expression of selected KRAB‐ZNFs in breast cancer compared to normal mammary epithelium. (A,B) Bars indicate mean expression for three technical replicates ± SD. (C) RT‐qPCR expression of selected KRAB‐ZNFs in lung cancer tissue samples compared to paired normal tissue. *P*‐values were calculated by Wilcoxon test. (D) RT‐qPCR expression of selected KRAB‐ZNFs in breast cancer tissue compared to normal tissue samples. *P*‐values were calculated using the Mann–Whitney *U‐*test. (E) Immunohistochemistry staining for selected KRAB‐ZNFs in lung and breast tissue microarray cancer samples from The Human Protein Atlas. (C,D) *P < 0.05, **P < 0.01, ***P < 0.001, ****P < 0.0001. Number of samples in each group and FC values are provided below the graphs.

To validate the expression results obtained from TCGA datasets, we analyzed 10 selected KRAB‐ZNFs in the panels of lung and breast cancer cell lines (see point 2.3) and tissues (see point 2.2, Table 1). Specifically, we examined the expression of chosen KRAB‐ZNFs in 16 different lung cancer cell lines and nine breast cancer cell lines representing different tumor subtypes. All of the selected cancer‐associated KRAB‐ZNFs were upregulated in the majority of lung cancer cell lines compared to the normal human bronchial epithelium cell line (NHBE) (Fig. 2A). As for breast cancer, we found that the expression level of most of the examined KRAB‐ZNFs was elevated at least twofold in almost every cell line in comparison with the normal mammary epithelial cell line, MCF12A (Fig. 2B). The only exemption was *ZNF789* that showed upregulation in one cell line, MDA‐MD‐M468.

In the next step, we evaluated KRAB‐ZNFs expression in lung and breast cancer tissue panels (Table 1). We compared the expression between 58 matched pairs of lung cancer and adjacent normal tissues and five normal breast and 43 cancer unmatched tissues. It is noteworthy that for some of the samples, KRAB‐ZNF expression fell below the detection level, and thus, the number of samples used for analysis varied between factors (Fig. 2C,D). Single‐gene qPCR assays confirmed that the expression level of 10 selected KRAB‐ZNFs was at least two times more abundant in lung tumor samples than in matched normal tissues (Fig. 2C). The biggest difference was observed in the case of *ZNF714* expression (FC = 13.1, *P* < 0.0001 as assessed by Wilcoxon test) and the lowest for *ZNF485* (FC = 2.2, *P* < 0.0001). In the breast cancer tissue panel (normal = 5, tumor = 43), we found that four KRAB‐ZNFs (*ZNF205*,* ZNF273*,* ZNF485*,* ZNF695*) were significantly upregulated in tumor tissue (Fig. 2D). The highest difference was observed for *ZNF695* (FC = 8.2, *P* < 0.001 as assessed by Mann–Whitney *U*‐test) and the smallest for *ZNF205* (FC = 3.0, *P* < 0.01). We also observed higher expression of *ZNF320* and *ZNF789* in tumors, but these data failed to reach statistical significance. Mean expression of *ZNF714* was similar in normal and tumor samples, whereas *ZNF525*,* ZNF643*, and *ZNF707* fell below the detection level.

These results were further confirmed on a protein level. We investigated cancer‐associated KRAB‐ZNFs in The Human Protein Atlas (www.proteinatlas.org) (Uhlen *et al*., 2015) that contains antibody‐based images of selected cancer types. In lung cancer tissue samples, KRAB‐ZNF staining was positive for at least four analyzed tumor samples (ZNF205 – 4/12, ZNF320 – 10/10, ZNF485 – 11/11, ZNF643 – 10/11, ZNF695 – 8/9, ZNF 707 – 10/11) and at least one sample from each set presented nuclear localization of a given factor (Fig. 2E). There were no data published for ZNF273, ZNF525, and ZNF714; and ZNF789 as staining was not detected in any of the analyzed samples. For breast cancer, the number of KRAB‐ZNF‐positive samples was slightly different: (ZNF205 – 6/11, ZNF320 – 10/10, ZNF485 – 11/11, ZNF643 – 11/11, ZNF695 – 12/12, ZNF 707 – 10/10, and ZNF789 – 2/12). No data were available for ZNF273, ZNF525, and ZNF714 (Fig. 2E).

### 3.3 Most of the cancer‐associated KRAB‐ZNF isoforms become simultaneously upregulated in tumors compared to normal samples

**Figure 3:**
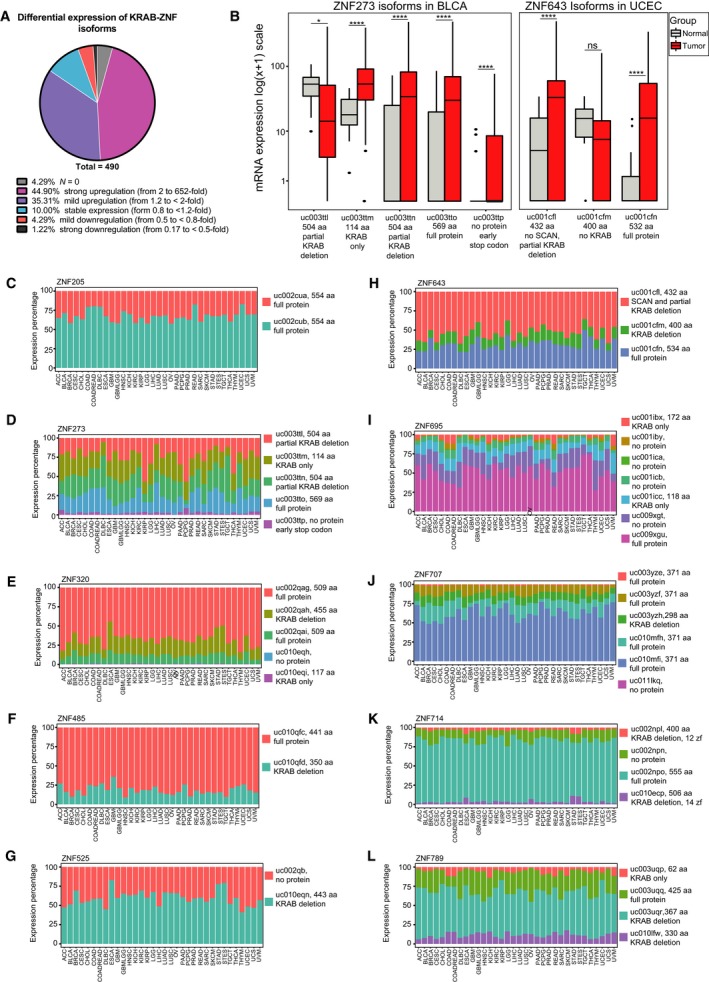
Expression of KRAB‐ZNF splicing isoform in TCGA datasets. (A) Pie chart depicting the percentage of isoforms that appeared statistically significant (*n *= 490) in our differential expression analysis comparing normal and tumor tissues. The isoforms fell into six categories: with null expression in normal samples, strongly and mildly upregulated in tumor samples, with stable expression, mildly and strongly downregulated in tumor samples. (B) The expression of only two pairs of isoforms is switched between normal and tumor samples. The boxplots represent the isoforms of *ZNF273* in BLCA (N: 19, T: 408) and *ZNF643* in UCEC (N: 11, T: 370). Statistical significance was calculated based on *t*‐test with FDR correction: *P < 0.05, ****P < 0.0001. (C–L) Plots demonstrate percentages of total expression of each of the listed splicing variants of 10 selected cancer‐associated KRAB‐ZNFs across 34 TCGA cohorts.

The complexity of the KRAB‐ZNF factors family is linked not only to the large number of its members but also to the high variety of splicing isoforms. As aberrant splicing is a frequent event in carcinogenesis, we wanted to explore the isoform signature for cancer‐associated KRAB‐ZNFs in TCGA datasets. First, we took into consideration the possibility of variant switch between normal and cancer tissue. Out of 600 comparisons between normal (*n* ≥ 9 per cohort) and cancer tissues (40 isoforms for 10 genes in 15 cohorts), 490 (81.7%) appeared statistically significant in our analysis (*P* < 0.05 as assessed by t‐test with FDR correction). We divided these variants into six groups based on their status of differential expression. As expected, we found that a majority of splicing variants (84.5%) were overexpressed in cancer tissues compared to their normal counterparts (Fig. 3A). Out of 490 significant isoforms, 21 variants (4.3%) showed expression only in cancer tissues, 220 variants (44.9%) were strongly overexpressed in cancer compared to normal (≥ 2‐fold overexpression, with the highest level reaching 652‐fold change), and 173 variants (35.3%) showed mild overexpression (FC < 2 and ≥ 1.2). It is of note that 21 isoforms that fell below the detection threshold in normal samples included mainly truncated and nonsense variants of *ZNF695*. The remaining 15.5% isoforms displayed either relatively stable expression (FC < 1.2 and ≥ 0.8 observed in 49 out of 490 variants, 10.0%), mild downregulation (FC < 0.8 and ≥ 0.5 in 21 variants, 4.3%), or strong downregulation (FC from 0.17 to < 0.5 in six variants, 1.2%).

In general, we observed that the expression of the splicing isoforms followed a similar pattern in cancer and normal samples. Thus, to address the question regarding the potential variant switch between cancer and normal tissues, we focused on the expression profiles of the isoforms downregulated in cancer tissues (*n* = 27). These isoforms fell into three categories. The first category represented 14 isoforms that belong to five KRAB‐ZNFs with higher expression in healthy tissues, out of which only *ZNF273* and *ZNF789* showed significant downregulation in PRAD and KICH, respectively. The second category comprised 11 isoforms, whose expression was not predominant in the given tissue (i.e., these isoforms have a low or medium expression level), which indicates a low impact on cell function. Finally, the third category harbored two isoforms, whose expression was switched to other isoforms (Fig. 3B). This included *ZNF273* in BLCA and *ZNF643* in UCEC. *ZNF273*, the isoform with a 5′ partial deletion of the KRAB domain, was switched to three other isoforms, which could be translated to a full‐length protein, a variant with a C‐terminal partial deletion of the KRAB domain, and a protein devoid of the zinc finger domain. *ZNF643*, the shortest variant, lacking the SCAN and KRAB domains, was switched to two longer isoforms translated into a 534 aa full‐length protein and a 432 aa protein with a C‐terminal deletion of the SCAN domain and partial deletion of the KRAB domain. Thus, these observations indicate that variant switch is a sporadic event in the case of the analyzed KRAB‐ZNFs.

Next, we compared the expression pattern of the splicing isoforms across 34 cohorts (apart from the cohorts used for the comparative analysis, we used additional datasets, including: ACC, CESC, COAD, COADREAD, DLBC, GBM, GBMLGG, LGG, OV, PAAD, PCPG, READ, SARC, SKCM, STAD, TGCT, THYM, UCS, and UVM). Although the exact expression values differed between various cancer types, the relative expression profile remained uniform across multiple cancers (Fig. 3C–L). However, there were some exemptions to this rule. Four out of five *ZNF273* variants (Fig. 3D) occurred in mixed configuration across all cohorts, but all of them showed a relatively high and equal expression level. The isoform expression pattern differed as well in the case of *ZNF525* (in DLBC, LIHC, THYM, UCEC, and UCS, Fig. 3G), and *ZNF643* (in GBMLLG, LGG, and TGCT, Fig. 3H). There was also some variability in the configuration of isoforms in the case of *ZNF695* (Fig. 3I) and *ZNF707* (Fig. 3J). Nevertheless, the most abundant isoform did not change. Similarly, in the case of *ZNF714* (Fig. 3K) and *ZNF789* (Fig. 3L), the variability occurred only among the two isoforms with the lowest expression. The most prevalent isoform expressed in the majority of tumors was the isoform encoding the longest protein. This is not the case for *ZNF525* (Fig. 3G) that was expressed in two isoforms: one lacking the KRAB domain and the other that is not translated into protein. Also, in the case of *ZNF643* (Fig. 3H), the isoforms with the highest expression level encoded a protein without the SCAN domain and with partial deletion of the KRAB domain, whereas the most abundant *ZNF789* variant contained no KRAB domain (Fig. 3L). It is of note, however, that in these two last instances, mRNA coding the full‐length protein were also expressed at a relatively high level.

### 3.4 Expression of cancer‐associated KRAB‐ZNFs correlates with histology, molecular subtypes, and patient survival in lung tumors

**Figure 4:**
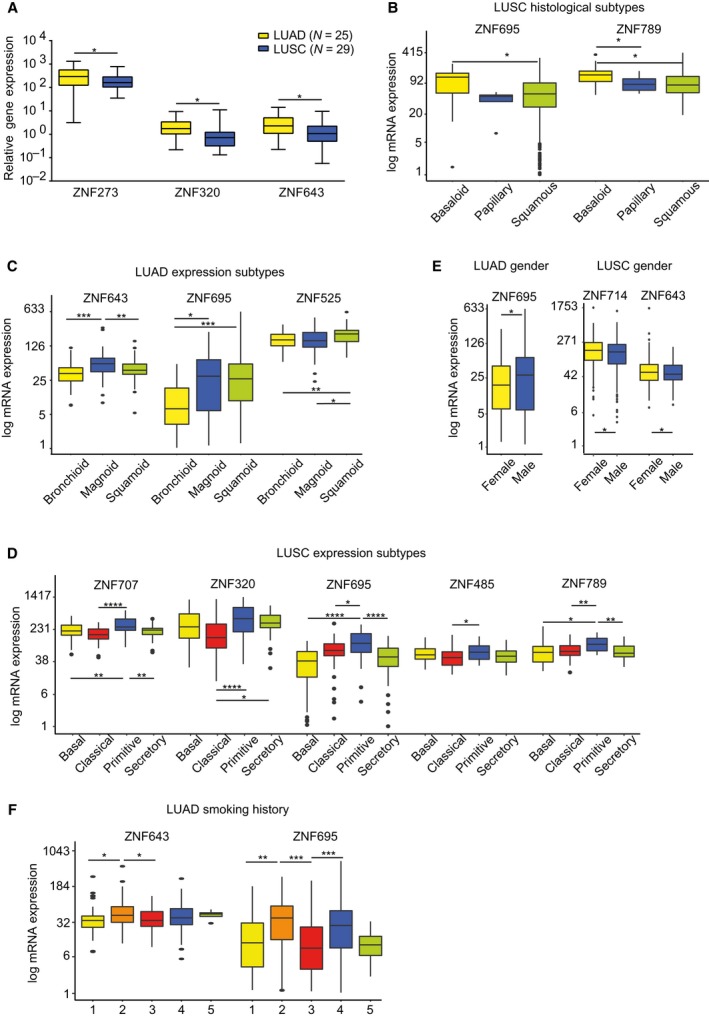
Cancer‐associated KRAB‐ZNFs correlate with various clinical parameters in LUAD and LUSC. (A) Single‐gene qPCR analysis in lung cancer tissue panel shows that *ZNF273*,*ZNF320*, and *ZNF643* have significantly higher mRNA level in LUAD (*n* = 25) compared to LUSC (*n* = 29). (B) In TCGA LUSC histological subtypes, *ZNF695* and *ZNF789* demonstrate higher expression in the basaloid (*n* = 15) subtype compared to other subtypes: papillary (*n* = 6) and/or squamous not otherwise specified (*n *= 467). (C) Boxplot representation of mRNA expression level of *ZNF525*,*ZNF643*, and *ZNF695* in TCGA LUAD molecular subtypes: squamoid (*n *= 78), bronchoid (*n* = 89), and magnoid (*n* = 63). (D) Boxplot representation of mRNA expression level of *ZNF707*,*ZNF320*,*ZNF695*,*ZNF485*, and *ZNF789* in TCGA LUSC molecular subtypes: basal (*n* = 42), classical (*n* = 65), primitive (*n* = 27), and secretory (*n* = 43). (E) The expression of *ZNF695* in TCGA LUAD, as well as *ZNF714* and *ZNF643* in TCGA LUSC correlate with gender. LUAD: female (*n* = 277), male (*n *= 239); LUSC: female (*n* = 130), male (*n* = 370). (F) *ZNF643* and *ZNF695* mRNA is more abundant in smokers vs nonsmokers or reformed smokers according to the TCGA dataset. 1: lifelong nonsmoker (*n* = 76), 2: current smoker (*n* = 119), 3: current reformed smoker for > 15 years (*n* = 135), 4: current reformed smoker for ≤ 15 years (*n* = 168), 5: current reformed smoker, duration not specified (*n* = 4). (A–F) *P < 0.05, **P < 0.01, ***P < 0.001, ****P < 0.0001 as assessed by *t*‐test with FDR correction.

Next, we wanted to explore the clinical significance of KRAB‐ZNF expression in tumor tissues using TCGA datasets and the independent validation set of lung cancer tissues (Table 1). To this end, we compared the expression of cancer‐associated KRAB‐ZNFs with various clinicopathological features, including histology, TNM classification, gender, survival, and smoking history (Table 2). We first utilized the data available for the lung cancer tissue panel (Table 1). We found that *ZNF273*,* ZNF320*, and *ZNF643* showed higher expression (~ 2‐fold) in LUAD than LUSC (*ZNF273*: FC = 1.85, *P* = 0.018; *ZNF320*: FC = 1.9, *P* = 0.042; *ZNF643*: FC = 1.84, *P* = 0.044, as assessed by *t*‐test with Tukey HSD correction, Fig. 4A). No other associations were found between KRAB‐ZNF expression and tumor size, differentiation, lymph node status, patient gender, or survival (Table 2).

**Table 2:** KRAB‐ZNF expression correlates with various clinicopathological parameters in lung cancer

Gene	Subtype	Subtype value	Mean ± SEM, Patients’ No.	*P* adjusted (*t*‐test)
*ZNF695*	LUSC histological subtypes	Basaloid	107.1 ± 17.1, *n* = 15	
		Papillary	34 ± 10, *n* = 6	vs Basaloid *P* = 0.045
		Squamous NOS	62.5 ± 2.5, *n* = 467	vs Basaloid *P* = 0.016
*ZNF789*		Basaloid	151.1 ± 22.1, *n* = 15	
		Squamous NOS	99.6 ± 2.73, *n* = 467	vs Basaloid *P* = 0.009
*ZNF525*	LUAD expression subtypes	Squamoid	220.7 ± 10.9, *n* = 78	
		Bronchioid	176,4 ± 6.4, *n* = 89	vs Squamoid *P* = 0.002
		Magnoid	184.5 ± 11.8, *n* = 63	vs Squamoid *P* = 0.03
*ZNF643*		Squamoid	45.3 ± 2.6, *n* = 78	vs Magnoid *P* = 0.005
		Bronchioid	36.9 ± 2, *n* = 89	vs Magnoid *P* < 10^−4^
		Magnoid	62 ± 6.1, *n* = 63	
*ZNF695*		Squamoid	40.5 ± 8.8, *n* = 78	vs Bronchioid *P* = 0.001
		Bronchioid	11.11 ± 1.3, *n* = 89	
		Magnoid	35.5 ± 5.31, *n* = 63	vs Bronchioid *P* = 0.01
*ZNF707*		Squamoid	203.5 ± 9.9, *n* = 78	*P* = 0.046
		Magnoid	249.5 ± 19.7, *n* = 63	
*ZNF714*		Squamoid	243.1 ± 20.7, *n* = 78	*P* = 0.03
		Magnoid	177.8 ± 17.5, *n* = 63	
*ZNF273*	LUSC molecular subtype	Basal	193.6 ± 16.8, *n* = 42	vs Primitive *P* = 0.02
		Classical	244.8 ± 18.8, *n* = 65	vs Secretory *P* = 0.006
		Primitive	289.9 ± 34.9, *n* = 27	
		Secretory	158 ± 11.7, *n* = 43	vs Primitive *P* = 0.0005
*ZNF320*		Basal	357.6 ± 45.6, *n* = 42	
		Classical	233.1 ± 27.1, *n* = 65	vs Primitive *P* < 10^−4^
		Primitive	527.3 ± 81.4, *n* = 27	
		Secretory	388.2 ± 33.4, *n* = 43	vs Classical *P* = 0.025
*ZNF695*		Basal	41 ± 5.2, *n* = 42	vs Primitive *P* < 10^−4^
		Classical	77.6 ± 6.5, *n* = 65	vs Primitive *P* = 0.009
		Primitive	116.5 ± 16.2, *n* = 27	
		Secretory	54.9 ± 6.8, *n* = 43	vs Primitive *P* < 10^−4^
*ZNF707*		Basal	232.5 ± 15.5, *n* = 42	vs Primitive *P* = 0.007
		Classical	186.1 ± 8.6, *n* = 65	vs Primitive *P* < 10^−4^
		Primitive	309.3 ± 29.5, *n* = 27	
		Secretory	220.8 ± 11.8, *n* = 43	vs Primitive *P* = 0.001
*ZNF789*		Basal	74.2 ± 8.4, *n* = 42	vs Primitive *P* = 0.01
		Classical	74.4 ± 4, *n* = 65	vs Primitive *P* = 0.007
		Primitive	104.2 ± 8.2, *n* = 27	
		Secretory	69.6 ± 4.6, *n* = 43	vs Primitive *P* = 0.003
*ZNF643*	LUSC gender	Female	77.7 ± 13.2, *n* = 130	*P* = 0.02
		Male	58.3 ± 1.8, *n* = 370	
*ZNF714*		Female	211.84 ± 17.6, *n* = 130	*P* = 0.049
		Male	179.6 ± 7.47, *n* = 370	
*ZNF695*	LUAD gender	Female	29.5 ± 2.3, *n* = 277	*P* = 0.011
		Male	41.1 ± 4.1, *n* = 239	
*ZNF643*	LUAD smoking history	Lifelong Nonsmoker	41.6 ± 4.3, *n* = 76	vs Current smoker *P* = 0.04
		Current smoker	57.4 ± 4.8, *n* = 119	
		Current reformed smoker for > 15 years	40.6 ± 1.8, *n* = 135	vs Current smoker *P* = 0.004
*ZNF695*		Lifelong Nonsmoker	23.7 ± 3.9, *n* = 76	vs Current smoker *P* = 0.002
		Current smoker	51.9 ± 4.9, *n* = 119	
		Current reformed smoker for > 15 years	18.3 ± 2.4, *n* = 135	vs Current smoker *P* < 10^−4^
		Current reformed smoker for < 15 years	42.9 ± 5.2, *n* = 168	vs Current reformed smoker for > 15 year *P* = 0.0004

We further analyzed the association between KRAB‐ZNF expression patterns and clinical parameters in LUAD and LUSC datasets from the TCGA project. We observed that the majority of significant differences in KRAB‐ZNF expression were related to histology (as noted in the tissue panel), molecular subtypes, and patient survival. In LUSC histological subtypes, we found that *ZNF695* and *ZNF789* had higher expression in the basaloid subtype than in the papillary and/or squamous subtypes, or in subtypes that were not otherwise specified (Fig. 4B). More associations between KRAB‐ZNF expression and clinical parameters were found in relation to molecular subtypes. We observed that in the LUAD mRNA subtypes, *ZNF525* had the highest expression in the squamoid subtype (magnoid and bronchioid), *ZNF643* expression was highest in the magnoid subtype, while *ZNF695* had the lowest expression in the bronchioid subtype (Fig. 4C). In LUSC, the highest KRAB‐ZNF expression was observed in the primitive subtype, particularly with respect to the classical subtype (Fig. 4D). This relation was true for *ZNF320*,* ZNF485*,* ZNF695*,* ZNF707*, and Z*NF789*. In general, no specific trend of association was found between KRAB‐ZNF expression and TNM classification or tumor stage both in LUAD and LUSC. Some associations were observed in the case of gender. In LUAD, *ZNF695* expression was higher in males, whereas in LUSC, *ZNF643,* and *ZNF714* expression was higher in females (Fig. 4E). Also, in LUAD, smoking history correlated with expression of *ZNF525*,* ZNF643*, and *ZNF695* (Fig. 4F). In all cases, the expression level in tumors from current smokers was significantly higher than in reformed smokers or nonsmokers. No such associations were observed in LUSC.

**Figure 5:**
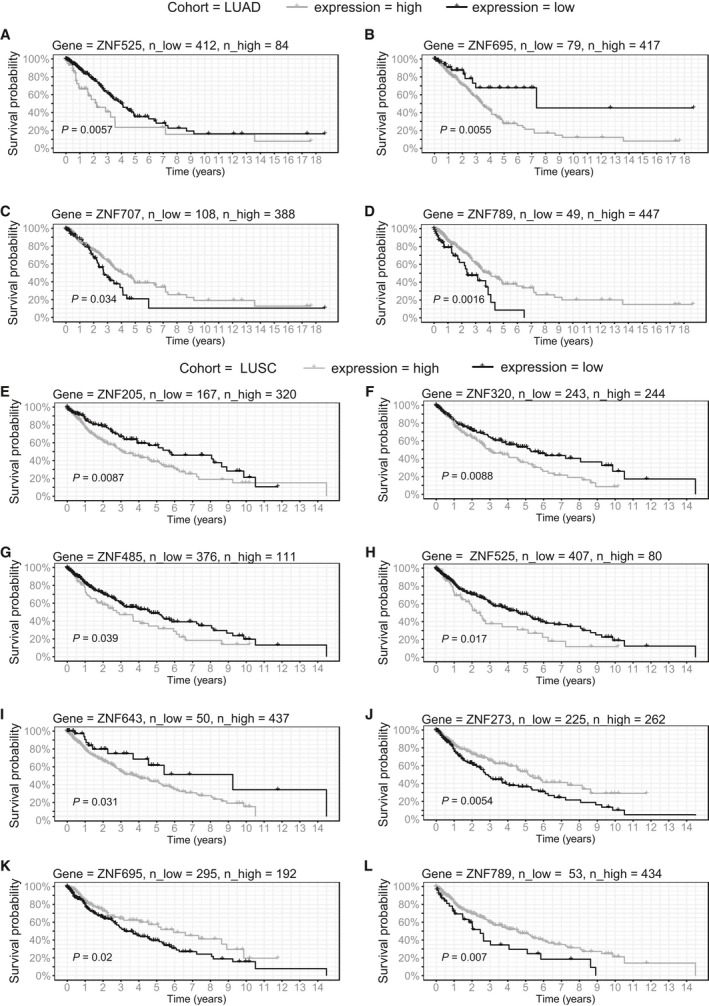
Survival analysis in TCGA LUAD and LUSC (E–L) patients. Kaplan–Meier curves represent *ZNF707* (A), *ZNF789* (B), *ZNF525* (C), and *ZNF695* (D) association with patient survival in LUAD and *ZNF205* (E), *ZNF320* (F), *ZNF485* (G), *ZNF525* (H), *ZNF643* (I), *ZNF273* (J), *ZNF695* (K), and *ZNF789* (L) association with patient survival in LUSC. KRAB‐ZNFs expression was transformed into binary high/low groups based on maximally ranked statistics. *P*‐values assessed by log‐rank test and number of patients in each group is provided in the figure.

Finally, we looked at the potential correlation of the KRAB‐ZNF expression with patient survival. In our log‐rank analysis, we used maximally selected rank statistics to identify the optimal cutoff point dividing the expression of each of the KRAB‐ZNFs into two groups with high and low mRNA levels. In LUAD patients, longer survival was observed in groups with low expression of *ZNF525* (*P* = 0.006, hazard ratio = 1.83) and *ZNF695* (*P* = 0.006, hazard ratio = 2.4) (Fig. 5A,B) and high expression of *ZNF707* (*P* = 0.034, hazard ratio = 0.66) and *ZNF789* (*P* = 0.002, hazard ratio = 0.5) (Fig. 5C,D). In LUSC patients, high mRNA levels of the majority of KRAB‐ZNF factors correlated with poor prognosis (Fig. 5E–I): *ZNF205* (*P* = 0.009, hazard ratio = 1.6), *ZNF320* (*P* = 0.009, hazard ratio = 1.5), *ZNF485* (*P* = 0.039, hazard ratio = 1.4), *ZNF525* (*P* = 0.017, hazard ratio = 1.6), and *ZNF643* (*P* = 0.031, hazard ratio = 1.9). In contrast, better prognosis was observed in the patients with high expression of *ZNF273* (*P* = 0.005, hazard ratio = 0.6), *ZNF695* (*P* = 0.02, hazard ratio = 0.7), and *ZNF789* (*P* = 0.007, hazard ratio = 0.6) (Fig. 5J–L).

### 3.5 Expression of cancer‐associated KRAB‐ZNFs correlates with histology, receptor status, molecular subtypes, and patient survival in BRCA

**Figure 6:**
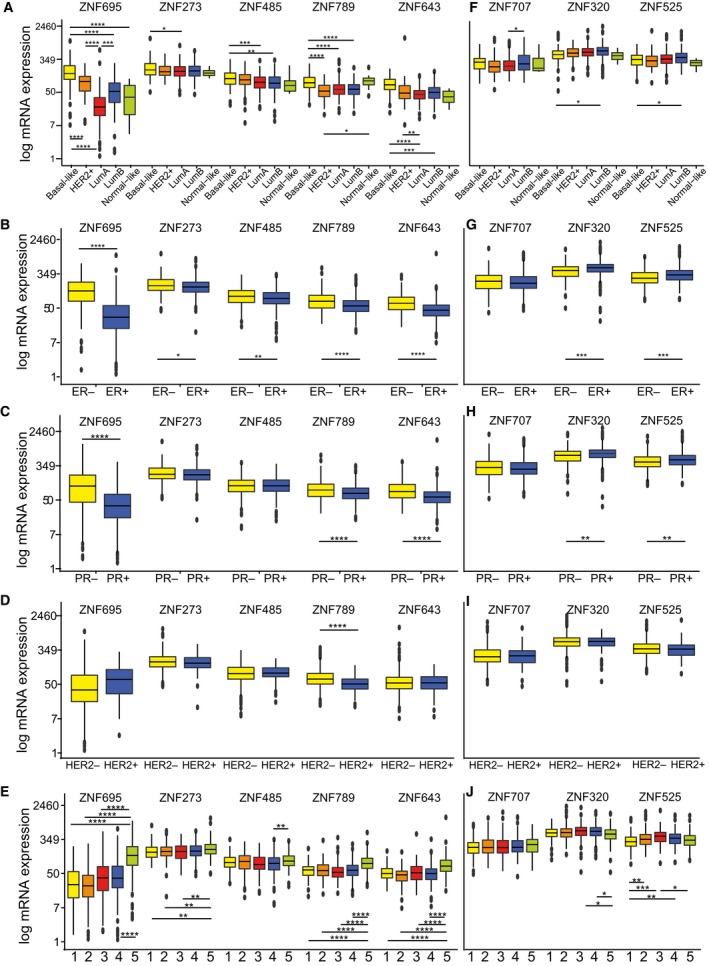
Cancer‐associated KRAB‐ZNF expression correlates with various clinical and molecular parameters in TCGA BRCA patients including: (A, F) histological subgroups: basal‐like (*n* = 98), HER2+ (*n* = 58), luminal A (*n* = 233), luminal B (*n* = 129), normal‐like (*n* = 8); (B, G) estrogen receptor (ER) status: ER+ (*n* = 603), ER− (*n* = 179); (C, H) progesterone receptor (PR) status: PR+ (*n* = 526), PR− (*n* = 253); (D, I) HER2 receptor status: HER2+ (*n* = 115), HER2− (*n* = 654); (E, J) methylation clusters: cluster 1: (*n* = 137), 2: (*n* = 174), 3: (*n* = 101), 4: (*n* = 246), 5: (*n* = 295). (A–J) *P < 0.05, **P < 0.01, ***P < 0.001, ****P < 0.0001 as assessed by *t*‐test with FDR correction.

**Figure 7:**
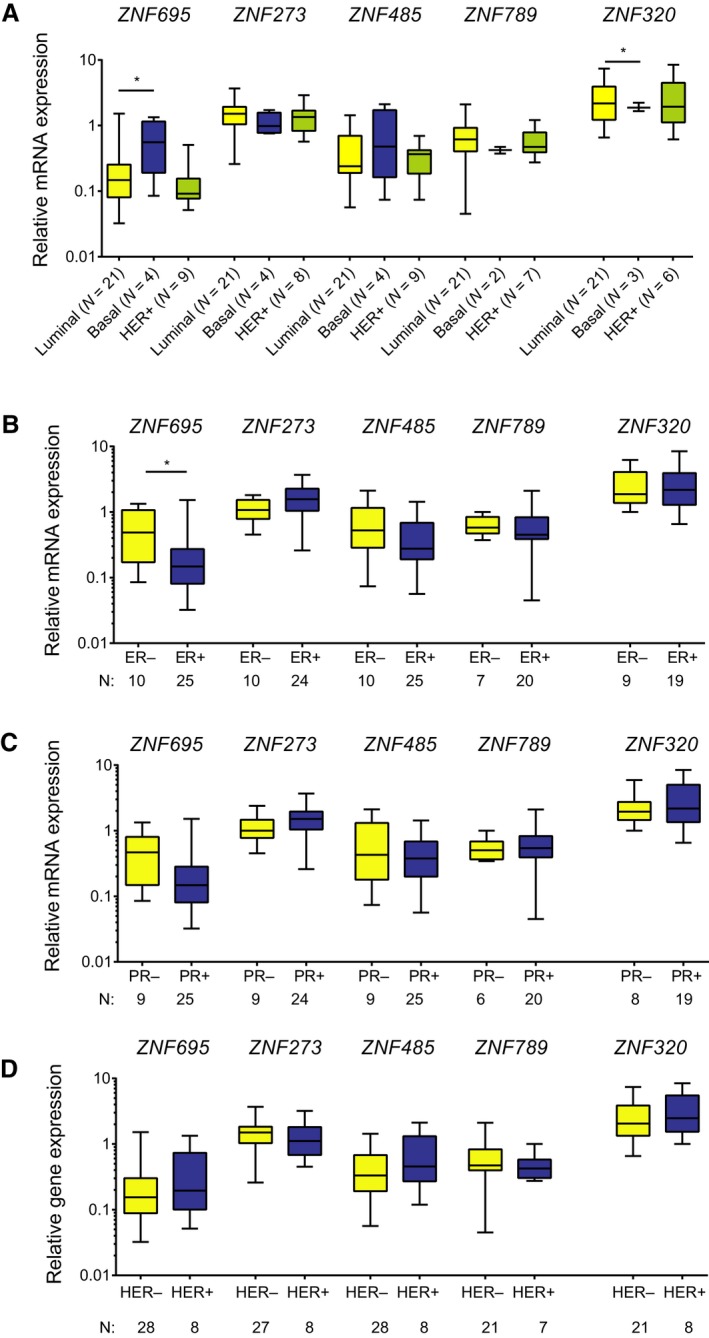
Cancer‐associated KRAB‐ZNF expression correlates with various clinical and molecular parameters in an independent panel of BRCA tissues. This includes histological subgroups (A), estrogen receptor (ER) status (B), progesterone receptor (PR) status (C), and HER2+ receptor status (D). *P < 0.05 as assessed by unpaired *t*‐test. The number of patients in each group is provided in the figure.

We further examined the clinical significance of KRAB‐ZNF expression in breast cancer. We found multiple interdependencies; however, based on the expression of KRAB‐ZNF in relation to histological and molecular subtypes, we were able to appoint two major categories (Fig. 6). The first one contained KRAB‐ZNFs with the highest expression in basal‐like tumors and included *ZNF695*,* ZNF643*,* ZNF485*, and *ZNF273* (Fig. 6A, Table 3). Significantly higher expression in basal‐like tumors in relation to other cancer types (excluding normal‐like) was also observed in the case of *ZNF789*. As expected, high expression of these factors also correlated with negative staining for estrogen receptor (ER) and progesterone receptor (PR) in the TCGA dataset (Fig. 6B,C, Table 4). However, *ZNF273* and *ZNF485* correlation with PR status did not reach statistical significance. As far as HER2 staining is concerned (Fig. 6D, Table 4), only *ZNF789* expression significantly correlated with HER2‐tumors in the TCGA dataset. Other factors showed either marginal difference or a trend of association with HER2+ tumors (*ZNF695*). In general, these observations (apart from some observations in the case of *ZNF273* and *ZNF789*) were also confirmed in the independent set of BRCA tissues (Fig. 7). However, the tissue panel results showed mainly a trend that did not reach statistical significance, possibly due to the lower number of samples. Finally, these factors (*ZNF273*,* ZNF485*,* ZNF695*,* ZNF643*, and *ZNF789*), together with *ZNF714*, had significantly increased mRNA levels in the DNA methylation cluster 5 identified for breast cancer (Fig. 6E, Table 5), which also coincides with the basal‐like subtype and the lowest overall DNA methylation level (The Cancer Genome Atlas Network, 2012).

**Table 3:** KRAB‐ZNF expression correlates with breast cancer histology status

Gene	Subtype value	Mean ± SEM	*P*‐value (adjusted)
*ZNF205*	Basal‐like	273.3 ± 16	*P* = 0.003
	Luminal A	334.2 ± 8.6	
*ZNF273*	Basal‐like	225.9 ± 14.7	*P* = 0.017
	Luminal A	188.1 ± 5.9	
*ZNF320*	Basal‐like	515.7 ± 25.8	*P* = 0.012
	Luminal B	620.9 ± 26.4	
*ZNF485*	Basal‐like	124.4 ± 6.5	
	Luminal A	97.2 ± 3	*P* = 0.0003 vs Basal
	Luminal B	97.4 ± 4.8	*P* = 0.002 vs Basal
*ZNF525*	Basal‐like	368.3 ± 16.9	*P* = 0.047
	Luminal B	446.6 ± 21.8	
*ZNF643*	Basal‐like	90.8 ± 4.2	
	HER2‐enriched	78.4 ± 21.2	*P* = 0.008 vs Luminal A
	Luminal A	49 ± 1.4	*P* < 10^−4^ vs Basal
	Luminal B	56.9 ± 2.5	*P* = 0.0003 vs Basal
*ZNF695*	Basal‐like	197 ± 15.6	
	HER2‐enriched	98 ± 7.8	*P* < 10^−4^ vs Basal
	Luminal A	29.8 ± 3	*P* < 10^−4^ vs Basal
			*P* < 10^−4^ vs HER2‐enriched
			*P* = 0.0007 vs Luminal A
	Luminal B	65.3 ± 4.8	*P* < 10^−4^ vs Basal
	Normal‐like	46.7 ± 13.9	*P* < 10^−4^ vs Basal
*ZNF707*	Luminal A	283.9 ± 10.5	*P* = 0.016
	Luminal B	347.3 ± 19.2	
*ZNF714*	Basal‐like	270.5 ± 18	
	HER2‐enriched	295. 4 ± 26.2	
	Luminal A	181.7 ± 6.8	*P* < 10^−4^ vs HER2‐enriched
			*P* < 10^−4^ vs Basal‐like
			*P* = 0.0002 vs Luminal B
	Luminal B	251 ± 14.6	
	Normal‐like	131.5 ± 25.2	*P* = 0.028 vs HER2‐enriched
*ZNF789*	Basal‐like	101.7 ± 5.5	
	HER2‐enriched	59.4 ± 4	*P* < 10^−4^ vs Basal
	Luminal A	67 ± 2.5	*P* < 10^−4^ vs Basal
	Luminal B	65.8 ± 2.8	*P* < 10^−4^ vs Basal
	Normal‐like	105.4 ± 19	*P* = 0.02 vs HER2‐enriched

**Table 4:** KRAB‐ZNF expression correlates with receptor status in breast cancer patients. ER, estrogen receptor; padj, adjusted *P*‐value; PR, progesterone receptor. Number of patients in each group: ER+ = 603, ER− = 179, N PR+ = 526, N PR− = 253, HER2+ = 115, HER2− = 653

Gene	Status	ER status		PR status		HER2 status	
		Mean ± SEM	*P*adj	Mean ± SEM	*P*adj	Mean ± SEM	*P*adj
*ZNF205*	−	263.9 ± 11.9	0.015	271.6 ± 9.3	0.025	296.6 ± 5.8	n.s.
	**+**	302.9 ± 5.6		304.8 ± 6.2		259.9 ± 10	
*ZNF273*	−	215.9 ± 9	0.028	205.6 ± 6.9	n.s.	200.2 ± 4	n.s.
	**+**	191.4 ± 3.7		192.1 ± 4		185.5 ± 7.7	
*ZNF320*	−	507.4 ± 16.6	0.0002	528 ± 14	0.002	583.8 ± 10	n.s.
	+	595.4 ± 9.9		297.2 ± 10.7		561.3 ± 18.6	
*ZNF485*	−	114.8 ± 4.4	0.004	104.6 ± 3.7	n.s.	103 ± 2.1	n.s.
	+	99.1 ± 2		101.8 ± 2.1		101.9 ± 3.8	
*ZNF525*	−	347.8 ± 12.3	0.0007	366.9 ± 11.1	0.01	404.8 ± 7.9	n.s.
	+	416. 2 ± 8.7		417.6 ± 9.5		389.2 ± 20.4	
*ZNF643*	−	84.7 ± 3.8	< 10^−4^	78.1 ± 2.9	< 10^−4^	63.7 ± 2.4	n.s.
	+	55.9 ± 2.3		54.7 ± 2.6		57.4 ± 2.6	
*ZNF695*	−	169.2 ± 10.3	< 10^−4^	136 ± 9	< 10^−4^	72.5 ± 3.9	n.s.
	+	46.8 ± 2.6		45.5 ± 2.1		77.8 ± 6.2	
*ZNF707*	−	299.2 ± 14.3	n.s.	296.6 ± 12	n.s.	291.1 ± 7.3	n.s.
	+	290.2 ± 7.6		290.7 ± 8.1		277.7 ± 15.6	
*ZNF714*	−	262.1 ± 13.7	0.004	250.5 ± 10.8	0.027	225.1 ± 5.9	n.s.
	+	216.4 ± 5.8		216 ± 6.2		249.5 ± 15.9	
*ZNF789*	−	93.8 ± 4.8	< 10^−4^	86.1 ± 3.8	< 10^−4^	76.4 ± 1.7	< 10^−4^
	**+**	67.7 ± 1.5		67.1 ± 1.5		53.7 ± 2.3	

**Table 5:** KRAB‐ZNF expression correlated with DNA methylation status in breast cancer. Cluster 1: *n* = 137, 2: *n* = 174, 3: *n* = 101, 4: *n* = 246, 5: *n* = 295

Gene	Methylation cluster	Mean	*P*‐value (adjusted)
*ZNF205*	1	271.3 ± 11.2	0.0007 vs 2
	2	336.7 ± 11.8	
	3	266.8 ± 12.9	0.001 vs 2
	4	293.5 ± 8.8	0.02 vs 2
	5	260.2 ± 13.3	< 10^−4^ vs 2
*ZNF273*	1	186.8 ± 8.2	0.006 vs 5
	2	289.2 ± 6.1	0.006 vs 5
	3	184.4 ± 8.2	0.008 vs 5
	4	200.3 ± 5.7	
	5	226.8 ± 5.7	
*ZNF320*	1	555.5 ± 16.5	
	2	586.3 ± 21.2	
	3	626.9 ± 27.6	0.016 vs 5
	4	602.9 ± 14.9	0.028 vs 5
	5	528.2 ± 19.3	
*ZNF485*	1	12.6 ± 4	
	2	102.9 ± 3.7	
	3	98.3 ± 5.6	
	4	97.8 ± 116	0.008 vs 5
	5	116 ± 4.6	
*ZNF525*	1	343.6 ± 12.8	
	2	422.4 ± 18.2	0.005 vs 1
	3	456.2 ± 22.4	0.0002 vs 1
	4	414.8 ± 12.3	0.008 vs 1
	5	374.2 ± 15.1	0.001 vs 3
*ZNF643*	1	55.9 ± 2.2	< 10^−4^ vs 5
	2	49.5 ± 1.9	< 10^−4^ vs 5
	3	62 ± 5.1	< 10^−4^ vs 5
	4	57 ± 2.2	< 10^−4^ vs 5
	5	95.5 ± 8.9	
*ZNF695*	1	48.3 ± 4.8	< 10^−4^ vs 5
	2	37 ± 3.1	< 10^−4^ vs 5
	3	58.9 ± 7.4	< 10^−4^ vs 5
	4	60.3 ± 4.4	< 10^−4^ vs 5
	5	180 ± 12.9	
*ZNF714*	1	197 ± 10.6	0.0001 vs 5
	2	198.8 ± 9.2	< 10^−4^ vs 5
	3	247.4 ± 17.3	
	4	227.6 ± 10	0.012 vs 5
	5	278.2 ± 14.9	
*ZNF789*	1	64.7 ± 2.4	< 10^−4^ vs 5
	2	66.7 ± 2.7	< 10^−4^ vs 5
	3	64.8 ± 4.8	< 10^−4^ vs 5
	4	68.1 ± 2.3	< 10^−4^ vs 5
	5	104 ± 4.3	

The second identified KRAB‐ZNF subgroup included *ZNF320*,* ZNF525*, and *ZNF707*, and its expression was significantly higher in luminal B tumors compared to that in basal‐like and/or luminal A tumors (Fig. 6F, Table 3). Apart from *ZNF707*, whose expression was not related to the receptor status, mRNA levels of *ZNF320* and *ZNF525* were significantly higher in ER+ and PR+ tumors (Fig. 6G,H, Table 4). In general, the expression of these KRAB‐ZNFs was not related to HER2 status (Fig. 6I, Table 4). Furthermore, *ZNF320* and *ZNF525* expression was low in DNA methylation clusters 5 and 1, while the highest expression was noted for cluster 3 (Fig. 6J, Table 5). Cluster 3 correlates with the luminal B subtype and the highest global DNA methylation profile (The Cancer Genome Atlas Network, 2012).

**Figure 8:**
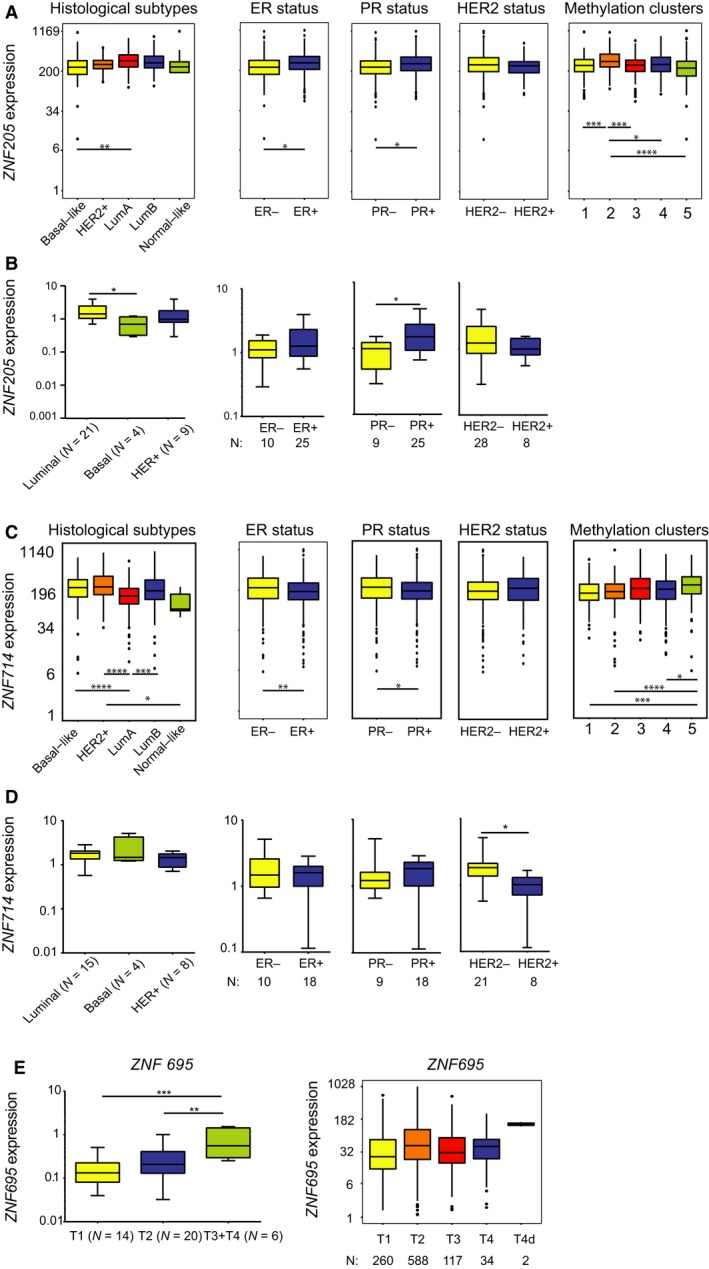
*ZNF205* and *ZNF714* correlate with various clinical and molecular parameters in BRCA samples. (A) *ZNF205* expression positively correlates with luminal A subtype, ER+, and PR+ tumors, while its expression is the highest in methylation cluster 2 in TCGA datasets. (B) *ZNF205* expression positively correlates with luminal A subtype and PR+ tumors in an independent set of breast cancer tissues. (C) *ZNF714* expression positively correlates with HER2+, ER−, and PR− tumors, while its expression is the highest in methylation cluster 5 in TCGA datasets. (D) *ZNF714* expression positively correlates with HER2− tumors in an independent set of breast cancer tissues. (E) *ZNF695* expression positively correlates with advanced tumor stage in an independent set of breast cancer tissues, but not in the TCGA dataset. (A, C) Basal‐like (*n* = 98), HER2‐enriched (*n* = 58), luminal A (*n* = 233), luminal B (*n* = 129), normal‐like (*n* = 8), ER+ (*n* = 603), ER− (*n* = 179), PR+ (*n* = 526), PR− (*n* = 253), HER2+ (*n* = 115), HER2− (*n* = 654), methylation cluster 1: (*n* = 137), 2: (*n* = 174), 3: (*n* = 101), 4: (*n* = 246), 5: (*n* = 295). (B, D, E) Number of samples provided in the figure. (A–E) *P < 0.05, **P < 0.01, ***P < 0.001, ****P < 0.0001 as assessed by *t*‐test with Tukey HSD correction (A, C, E) or unpaired *t*‐test (B, D).

The remaining cancer‐associated KRAB‐ZNFs did not show any characteristic features. In the TCGA dataset and tissue panel, *ZNF205* expression was relatively equal among the various histological subtypes, although higher expression was noted in the luminal compared to basal‐like tumors (Fig. 8A,B, Table 3). Also, significantly higher expression of *ZNF205* was noted in ER+ and PR+ tumors, and a trend of higher expression was observed in HER2‐ tumors (Table 4). The highest expression of *ZNF205* was seen in DNA methylation cluster 2 (Table 5). *ZNF714* expression (Fig. 8C, Table 4) was highest in HER2‐enriched tumors and lowest in luminal A, and as expected, in the ER−, PR−, and HER2+ tumors in the TCGA dataset. Surprisingly, in the BRCA tissue panel, high *ZNF714* expression correlated with ER−, PR+, and HER2− samples (Fig. 8D, Table 4).

**Figure 9:**
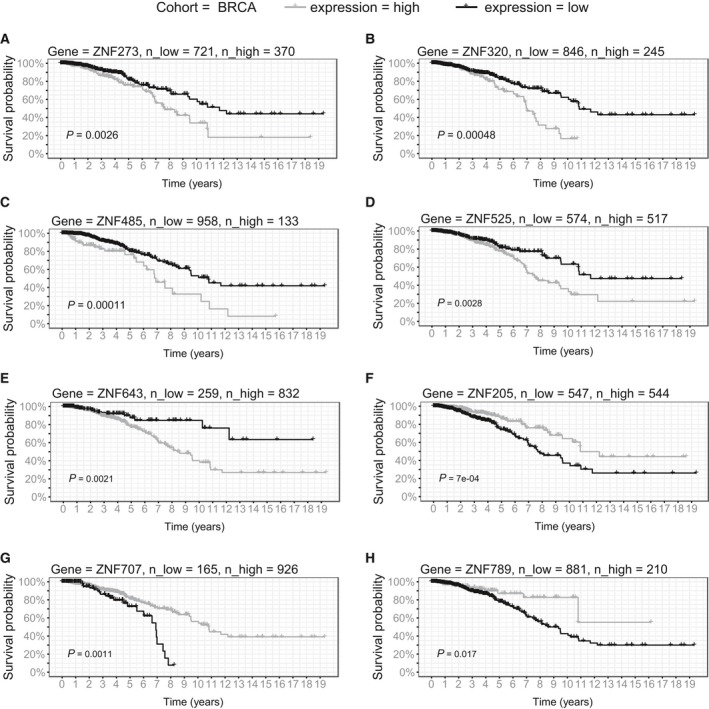
Survival analysis in TCGA BRCA patients. Kaplan–Meier curves represent *ZNF273* (A), *ZNF320* (B), *ZNF485* (C), *ZNF525* (D), *ZNF643* (E), *ZNF205* (F), *ZNF707* (G), and *ZNF789* (H) association with patient survival in LUSD. KRAB‐ZNFs expression was transformed into binary high/low groups based on maximally ranked statistics. *P*‐values assessed by log‐rank test and number of patients in each group is provided in the figure.

As far as other clinicopathological features are concerned, the expression of the majority of KRAB‐ZNFs did not present any distinct trend of association with TNM classification, tumor stage, or patient gender. Only *ZNF695* exhibited a positive correlation between expression and tumor size in the BRCA tissue panel, but not in the TCGA dataset (Fig. 8E). Finally, our survival analysis indicated that the expression of KRAB‐ZNFs may act as a risk factor. Patients with high expression of five out of 10 analyzed KRAB‐ZNFs presented significantly shorter overall survival than those with low expression (Fig. 9A–E). This included *ZNF273* (*P* = 0.003, hazard ratio = 1.8), *ZNF320* (*P* < 0.001, hazard ratio = 2.0), *ZNF485* (*P* < 0.0001, hazard ratio = 2.3), *ZNF525* (*P* = 0.003, hazard ratio = 1.8), and *ZNF643* (*P* = 0.002, hazard ratio = 2.4). In contrast, high expression was associated with better prognosis in the case of *ZNF205* (*P* < 0.001, hazard ratio = 0.5), *ZNF707* (*P* = 0.001, hazard ratio = 0.5), and *ZNF789* (*P* = 0.017, hazard ratio = 0.5) (Fig. 9F–H).

## 4 Discussion

The KRAB‐ZNF family is highly complex because it comprises a large number of homologous genes, gene isoforms, and pseudogenes. Such an enormous variety has so far hampered detailed molecular characterization of these proteins. While a growing number of studies reports KRAB‐ZNF involvement in multiple aspects of tumor biology, we aimed to provide a comprehensive portrayal of cancer‐associated KRAB‐ZNFs using publicly available TCGA pan‐cancer datasets. The results obtained from TCGA data were further validated in the independent sets of lung and breast cancer cell lines and tissues. Interestingly, we found many KRAB‐ZNF factors commonly downregulated and only a subset of a few KRAB‐ZNFs upregulated in multiple cancer types. This observation is in agreement with the results reported by Addison *et al*. (2015). Their study indicated that in the breast cancer TCGA datasets, the majority of KRAB‐ZNFs were downregulated, and only a small fraction was expressed at a higher level. These alterations were attributed to genomic changes involving nonsense mutations, genomic deletions, and amplifications (Addison *et al*., 2015). Similarly, we observed a small subgroup of KRAB‐ZNFs, overexpressed in multiple cancer types (Fig. 1A). KRAB‐ZNFs are known for their repressive function acting through epigenetic mechanisms, such as deposition of H3K9me3 and DNA methylation (Czerwinska *et al*., 2017; Ecco *et al*., 2017; Groner *et al*., 2010). It is well established that KRAB‐ZNFs bind to the sequences of transposable elements (TEs; Ecco *et al*., 2017). This leads not only to the restraint of TE expression and transposition (Jacobs *et al*., 2014), but also to the domestication of their regulatory sequences (e.g., promoters, enhancers) for the advantage of a cell (Ecco *et al*., 2016; Imbeault *et al*., 2017; Trono, 2015). It is noteworthy that the genes under the control of KRAB‐ZNF may undergo not only repression, but also activation (Busiello *et al*., 2017; Frietze *et al*., 2010; Lupo *et al*., 2013). At this stage, it is difficult to envisage how the cancer‐associated KRAB‐ZNFs affect tumor behavior, and further functional analyses are necessary to answer this question. From the set of upregulated KRAB‐ZNFs, only few were previously linked to cancer. Nevertheless, their exact molecular functions remain unknown. This includes *ZNF695* (Juarez‐Mendez *et al*., 2013; Li *et al*., 2015, 2017; Takahashi *et al*., 2015), *ZNF320* (Chernova *et al*., 2001), *ZNF138* (Tommerup and Vissing, 1995), *ZNF200* (Peedicayil *et al*., 2010), *ZNF707* (Nesslinger *et al*., 2007), and *ZNF354A* (von Eyben, 2004). Interestingly, cancer‐related upregulation of *ZNF695*,* ZNF714*, and *ZNF138* mirrors the differential expression observed between pluripotent stem cells and differentiated cells (Oleksiewicz *et al*., 2017). This observation underlines the similarities between cancer and stem cell expression profiles.

We further focused our attention on the differential expression of splicing isoforms. We observed that the majority of variants was detected both in normal and cancer tissues, but as expected, they had a higher level in tumors. The upregulation of the isoforms in malignant tissue seemed to occur simultaneously rather than through a variant switch mechanism. The differential expression of KRAB‐ZNF splicing isoforms in cancer was reported only by Juarez‐Mendez and colleagues (Juarez‐Mendez *et al*., 2013), who demonstrated a specific increase of *ZNF695* variants in ovarian cancer compared to normal cells. Interestingly, we frequently observed increased expression of the variants that are not translated to a protein or are short forms devoid of KRAB or zinc finger domains. It is likely that this phenomenon occurs due to the utilization of similar activation mechanisms that augment the expression of all involved isoforms. However, additional work is required to test this hypothesis. Also, future studies are needed to elucidate the relevance of individual isoforms for the functioning of a tumor cell.

Out of all analyzed cancer‐associated KRAB‐ZNFs, *ZNF695* seems to be the most interesting factor. In general, it showed a striking upregulation in many examined tumor types compared to their normal counterparts. Moreover, *ZNF695* expression was higher in more aggressive basal‐like and HER2‐enriched breast cancers associated with early relapse and poor clinical outcome (Kennecke *et al*., 2010; Sorlie *et al*., 2001, 2003). This observation stays in agreement with the recent study by Li *et al*. (2015), who also revealed high levels of *ZNF695* in basal‐like and HER2‐enriched tumors in three large breast cancer cohorts. In their study, *ZNF695* was classified as one of 16 master regulators capable of differentiating luminal and nonluminal BRCA subtypes. *ZNF695* was also shown to upregulate cell cycle progression genes (e.g., *CDK1*,* PLK1*) cooperatively with other master regulators in nonluminal breast cancers (Li *et al*., 2015). Also, in lung cancer, *ZNF695* might be indirectly associated with proliferation. We observed that in LUAD *ZNF695* expression is significantly higher in bronchioid and magnoid mRNA subtypes that demonstrate overrepresentation of growth and proliferation pathways, respectively (Hayes *et al*., 2006). In LUSC, *ZNF695* revealed the highest level in the primitive mRNA subtype with overexpressed proliferation genes (Wilkerson *et al*., 2010). Furthermore, we have previously shown that *ZNF695* was upregulated in pluripotent stem cells compared to more specialized cell types, whereas its knockdown resulted in the loss of self‐renewal properties and differentiation of pluripotent stem cells (Oleksiewicz *et al*., 2017). Its involvement in the stem cell phenotype was particularly evident in breast cancers, in which *ZNF695* expression was the most abundant in basal‐like tumors, known for their high content of stem cells (Honeth *et al*., 2008; Park *et al*., 2010). Thus, it is tempting to speculate that *ZNF695* may contribute to cancer stem cell identity, at least in basal‐like breast tumors. Nevertheless, further studies are needed to validate such an association.

## 5 Conclusions

In conclusion, we performed a TCGA pan‐cancer expression analysis of the KRAB‐ZNF family of genes and showed that a small subset of its members was commonly upregulated in multiple cancer types. We further validated this observation in independent sets of breast and lung cancer tissues and cell lines. Next, we demonstrated that the expression of the majority of cancer‐associated KRAB‐ZNF splicing isoforms was simultaneously upregulated in tumors as compared to normal adjacent tissues. Furthermore, we found that the expression of KRAB‐ZNFs in breast and lung cancer tissues correlated with histological and molecular subtypes, as well as patient survival. Our observations indicated that these KRAB‐ZNFs may play an oncogenic role during carcinogenesis, which makes them potential biomarkers and/or targets of anticancer therapies. Nevertheless, further studies are needed to elucidate their exact molecular functioning.

## Author contributions

UO and MM contributed to the conceptualization of this article. UO, MM, RC, KK, PB, and TL involved in methodology and investigation. UO, MM, RC, KK, and PB performed software, validation, and formal analysis. UO and MM wrote and drafted the original manuscript. UO, MM, RC, KK, PB, TL, and AM wrote, reviewed, and edited the manuscript. UO, MM, KK, RC, and PB involved in visualization. UO, PB, and AM involved in supervision of the article. UO and PB contributed to funding acquisition.

## Conflicts of interest

The authors declare no conflict of interest.
